# Nodular Fasciitis Complicating a Staged Surgical Excision of Dermatofibrosarcoma Protuberans

**DOI:** 10.1155/2016/6074182

**Published:** 2016-11-27

**Authors:** C. Helen Malone, Brandon Goodwin, Richard F. Wagner, Vicente Resto, Brent Kelly

**Affiliations:** ^1^Department of Dermatology, The University of Texas Medical Branch, Galveston, TX 77555, USA; ^2^Department of Otolaryngology, The University of Texas Medical Branch, Galveston, TX 77555, USA

## Abstract

Dermatofibrosarcoma protuberans (DFSP) is an unusual spindle cell tumor with a high rate of local recurrence with traditional excision. Fortunately, Mohs micrographic surgery yields excellent cure rates for this neoplasm due to contiguous tumor spread and meticulous tumor mapping and margin analysis. We present the unique case of a patient treated with a modified Mohs technique with an analysis of the final margin with permanent sections, who developed a spindle cell neoplasm in the margins of her second stage excision consistent with nodular fasciitis. Distinguishing residual DFSP from a benign reactive process was an essential and challenging component of this patient's management.

## 1. Introduction

Dermatofibrosarcoma protuberans is a histologically and clinically low-grade soft tissue sarcoma with a high rate of recurrence following local excision. Current guidelines advocate wide local excision or Mohs micrographic surgery to achieve negative surgical margins. Pathologically confirmed complete margin control is currently recommended for treatment and is essential for local disease control.

Nodular fasciitis is a benign reactive process that mimics malignant tumors clinically, in imaging studies and on fine-needle aspiration cytology. To our knowledge, nodular fasciitis has no previously known association with dermatofibrosarcoma protuberans. We present the challenging case of a 52-year-old woman with a dermatofibrosarcoma protuberans on her right chin who underwent staged surgical excision with permanently processed en face margins. Nodular fasciitis complicated the interpretation of her final margins. The diagnosis of nodular fasciitis was critical in our patient, because it avoided additional surgical excision with marginal mandibulectomy, more extensive surgical reconstruction, and possible adjuvant therapy.

## 2. Case Presentation

**Figure 1:**
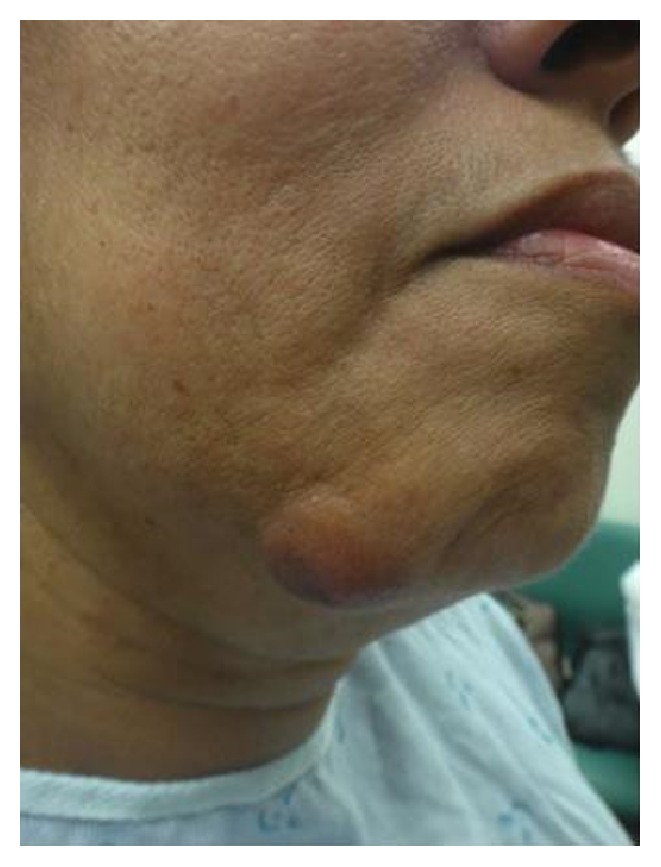
52-year-old African American woman with 4 cm firm but mobile erythematous nodule on the right side of her chin.

**Figure 2:**
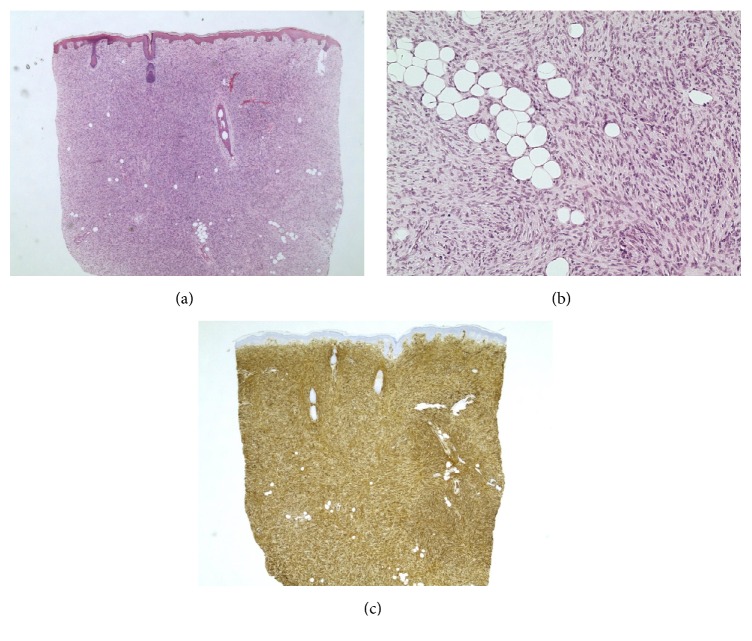
(a) 20x magnification of original punch biopsy specimen: a diffuse dermal proliferation of spindle cells extending into the subcutaneous fat. (b) 200x magnification of original biopsy specimen: a hypercellular dermal proliferation of spindle cells in a storiform pattern. Infiltration of fat in a “honeycomb” pattern is noted. (c) 20x magnification of original biopsy specimen: spindle cells stain diffusely positive for CD34.

A 52-year-old African American woman presented to the university dermatology department 100 miles from her home for a biopsy proven dermatofibrosarcoma protuberans (DFSP) on her right chin (Figure 1). The tumor had been present for approximately one year. Initial pathology was classic for DFSP with a dense storiform spindle cell proliferation infiltrating the underlying adipose tissue with diffusely positive staining for CD34, focal SMA staining, and negative staining for S100, AE1/3, and Factor XIIIa (Figures 2(a), 2(b), and 2(c)). Given the high recurrence rates with conventional excision and cosmetically sensitive location of the tumor, staged surgical excision with permanent processing en face margin evaluation was chosen as the treatment modality. The clinically palpable tumor was debulked sharply and submitted for pathology in the outpatient clinic. A second excision for margin evaluation included a 0.5 cm circumferential and deep margin, taken perpendicular to the wound edge and down to the supraperiosteal plane. Hatch marks denoting a clock face were placed at the 12, 3, 6, and 9 o'clock positions on the excision specimen and 5-0 prolene simple interrupted sutures were placed in the corresponding adjacent native skin. A corresponding map of the wound was drawn. A 20 cm^2^ fenestrated E-Z Derm® Porcine Xenograft was sewn in place over the defect with 4-0 monocryl suture prior to dressing placement. Wound care involved daily application of mupirocin 2% ointment to the xenograft and a protective dry dressing.

**Figure 3:**
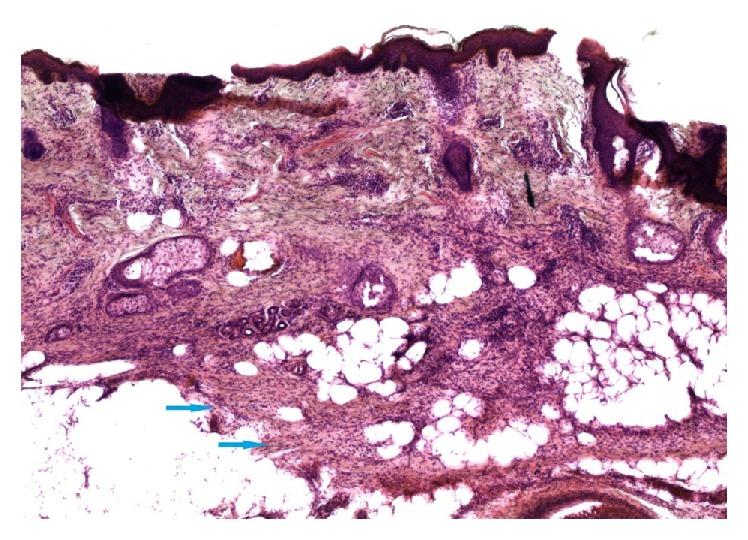
20x magnification of Stage 1 excision: focally positive margin with dense spindle cells infiltrating the subcutaneous tissue (arrows).

Careful joint review of the marginal tissue by the surgeons and dermatopathologists from the excision demonstrated residual tumor in the dermis (Figure 3) and muscle.

One week after the initial surgery, a second en face stage was taken from the wound areas still positive for DFSP with an additional 0.5 cm cutaneous margin extending deep to include the periosteum and submitted for permanent processing. A 30 cm^2^ fenestrated E-Z Derm Porcine Xenograft was sewn in place over the defect with 4-0 monocryl suture prior to dressing placement. Hatch marks on the excision with corresponding prolene suture in the adjacent native skin were also placed, and a wound map was drawn. Wound care was identical to earlier excision.

**Figure 4:**
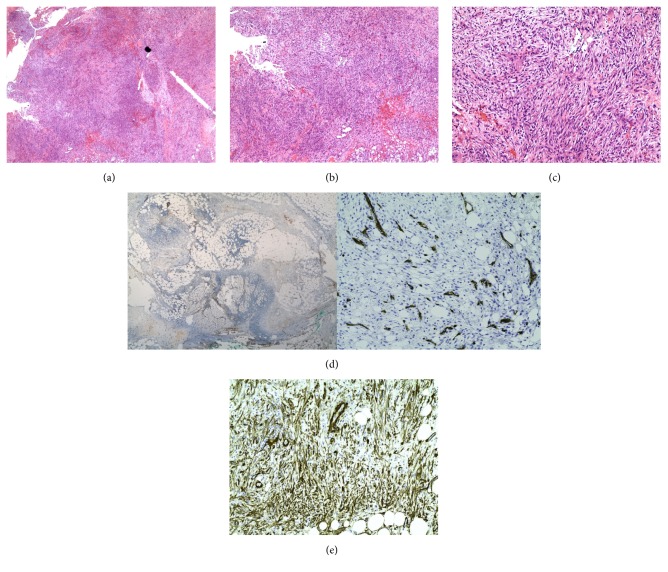
(a) 20x magnification of Stage 2 excision: a new diffuse deep dermal proliferation of spindle cells with extension into the subcutaneous tissue. Areas of hyper- and hypocellularity, myxoid stroma, and extravasated red blood cells can be noted at low power. (b) 100x magnification of Stage 2 excision: proliferation of spindle cells within a myxoid stroma with foci of inflammatory infiltrate including eosinophils and extravasated red blood cells. (c) 200x magnification of Stage 2 excision: “tissue culture” like appearance of fibroblasts within a myxoid stroma with extravasated red blood cells and acute inflammation. (d) 20x magnification of Stage 2 excision (left) and 100x magnification of Stage 2 excision (right): spindle cells stain negative for CD34. This immunohistochemical finding favors nodular fasciitis over residual DFSP. (e) 100x magnification of Stage 2 excision: spindle cells stain positive for SMA. This immunohistochemical finding favors nodular fasciitis over residual DFSP.

The histological findings from the second surgical stage were concerning for residual DFSP because an extensive spindle cell proliferation was noted in the dermal and periosteal margins of the tumor (Figures 4(a) and 4(b)). However, the spindle cell proliferation was distinctly different from the patient's original diagnostic DFSP biopsy and previous positive margins. The pathological examination revealed loosely arranged spindle cells with a “tissue culture” like appearance within a myxoid stroma (Figure 4(c)). The spindle cells were CD34 negative, and granulation tissue, focal hemorrhage, eosinophils, and multinucleated giant cells were present (Figures 4(d) and 4(e)).

After careful consideration, a diagnosis of nodular fasciitis was favored. The patient's final defect size was 4.6 × 6.5 cm with exposed bone at the base of the wound. A cervicofacial rotation-advancement flap was used for soft tissue reconstruction. She had no clinical evidence of tumor recurrence at her 12-month follow-up visit, and a computed tomography scan at that time was also negative.

## 3. Discussion

DFSP is a unique low-grade soft tissue sarcoma, characterized by a significant risk of local recurrence but minimal risk of metastasis or death [1, 2]. The typical clinical presentation is a firm nodule or plaque on the trunk or proximal extremities without adherence to underlying bone. Local recurrence rates range dramatically from 1 to 60%, but these rates are improved with Mohs micrographic surgery (MMS) with reported recurrence rates of <2%. The lung is the most common site of metastatic disease and rates of metastasis are low: ranging from 1 to 4% [1, 3]. The patient presented above is slightly unusual because her tumor involved the face, but she otherwise fit with the typical clinical presentation for DFSP.

Characteristic pathology findings of DFSP include a relatively monomorphic spindle cell proliferation in the dermis extending into the underlying subcutaneous tissue with several known histologic variants: pigmented (Bednar tumor), fibrosarcomatous, myofibroblastic, granular cell, and myxoid forms [4]. Classic DFSP is composed of a dense proliferation of uniform spindle cells in a cartwheel or storiform pattern with invasion of the subcutaneous tissue in a characteristic “honeycomb” pattern. Minimal cytologic atypia is present with minimal to no mitoses and diffuse staining for CD34 is evident [1–5]. Our patient's initial biopsy, debulking specimen, and first stage en face margins were typical of DFSP pathology.

The myxoid variant of DFSP and DFSP with fibrosarcomatous change (DFSP-FS) represents potentially confounding diagnoses in the setting of DFSP with nodular fasciitis. The myxoid variant is exceptionally rare and is characterized by a prominent myxoid stroma involving about 80% of the tumor. Other unusual features of this variant include a multinodular growth pattern, mast cells, weak CD34 staining in myxoid areas, and blood vessels. Local recurrence with the myxoid DFSP variant is thought to be slightly lower than DFSP with classic histology [4, 6]. Foci of fibrosarcomatous change involving at least 5% of the specimen are found in 10–20% of DFSP tumors [1–3]. In contrast to DFSP, fibrosarcomatous change is characterized by a dense proliferation of fusiform cells in a fascicular or herringbone pattern with significant cellular atypia, numerous mitosis, large nuclei, and negative staining for CD34 [1, 2]. A systematic review of 1422 patients with DFSP and 225 DFSP-FS found an increased risk of local recurrence (29%), metastasis (14%), and death (14%) with any proportion of fibrosarcomatous change. One should consider radiologic screening with chest computed tomography or magnetic resonance imaging to monitor for recurrence and metastatic disease in the setting of DFSP-FS [1]. Although in both myxoid and fibrosarcomatous areas of DFSP can both show loss of CD34 staining, the overall pattern of our patient's spindle cell proliferation in her final margin was more consistent with nodular fasciitis.

Nodular fasciitis is a benign process featuring fibroblast proliferation and pseudosarcomatoid features aptly first described as pseudosarcomatous fibromatosis [7, 8]. Several case reports describe nodular fasciitis with clinical presentations, imaging studies, and fine-needle aspiration cytology mimicking malignancy leading to misdiagnosis of breast cancer, sarcoma, and pleomorphic adenoma [7–10]. Although the pathogenesis eludes current knowledge, proposed mechanisms include response to traumatic injury or other reactive and inflammatory processes [7]. Well-defined masses of fibroblasts and myofibroblasts in a myxoid stroma create the classically described “tissue culture” like appearance of nodular fasciitis. In addition, nodular fasciitis can be infiltrative with numerous mitoses and focal red blood cell extravasation, but it does not have cytologic atypia or necrosis. Positive staining for vimentin and smooth muscle actin is expected, and negative staining for CD34, desmin, S100, and *β*-catenin is also typical [1].

Recurrence rate after complete excision ranges from 0 to 2% and spontaneous regression has also been described [11, 12]. USP6 FISH testing was negative in our patient which raises the possibility of nodular fasciitis-like granulation tissue as an alternative diagnosis. USP6 testing is negative in 10 to 14% of nodular fasciitis cases, so FISH testing is not a sensitive enough test to exclude the diagnosis of nodular fasciitis [13, 14]. Pseudoepitheliomatous hyperplasia that develops in skin cancer excision sites is an analogous problem, because it is a reactive process that can be mistaken for squamous cell carcinoma at the time of reconstruction [15]. We suspect that nodular fasciitis versus a similar reactive process developed during the week between the patient's first and second staged surgical excisions as a response to surgical trauma and possibly an inflammatory reaction to xenograft placement or topical antibiotic.

The diagnosis of a secondarily reactive entity rather than a DFSP tumor variant was more likely in our patient given the correlation between the clinical and pathologic findings. We used a staged excision technique with en face processing that permitted precise tumor mapping. There was minimal residual tumor present on the first stage en face margins. In contrast, the second stage en face margins showed a diffuse spindle cell proliferation that was highly unlikely to represent contiguous tumor growth from the small areas of tumor extension noted from the margins on the first stage.

Current National Comprehensive Cancer Network (NCCN) guidelines for treatment of DFSP strongly advocates excision with negative surgical margins and reexcision in the setting of positive margins. Options for excision include Mohs micrographic surgery with horizontal frozen sections, modified Mohs technique with an analysis of the final margin with permanent sections, and wide local excision with 2 to 4 cm margins including muscle fascia [3]. A case series of 35 patients with DFSP treated with a modified Mohs technique with paraffin tissue processing found no evidence of tumor recurrence with a median follow-up time of 30 months [16]. Our technique, while technically not Mohs surgery because the surgery and pathology were performed by different physicians, mimics “slow Mohs” in which both are performed by the same physician. In this instance the tissue was not frozen to produce horizontal frozen sections before permanent processing was performed. The distinction in terminology between slow Mohs and staged surgical excision techniques is important in the United States because in the latter, the pathology is billed separately by a different physician. If negative surgical margins are not feasible, radiation therapy is the preferred treatment modality although imatinib mesylate is an alternative option [1, 3]. Imatinib is a tyrosine kinase inhibitor, which is effective in patients with a COL1A1-PDGFB fusion gene found in 92% of patients with DFSP [1, 5, 17]. The NCCN suggests that imaging studies might be useful to detect recurrence in patients with high-risk disease [3]. A limitation of this case report is the short term follow-up, because most reported DFSP recurrences occur in the first three years after surgery [18]. Clinical exam and computed tomography scan imaging showed no evidence of tumor recurrence 12 months after the patient's excision.

## 4. Conclusion

Margin analysis for DFSP is an essential component of the treatment given the proclivity of DFSP for local recurrence. The presence of nodular fasciitis presented a considerable diagnostic challenge in determining a negative margin in this report of a staged surgical excision with permanent processing and en face margin analysis for DFSP. Loss of CD34 staining in combination with architecture distinct from myxoid and fibrosarcomatous variants of DFSP lent support to our diagnosis. In addition, precise tumor mapping in a tumor with known contiguous spread suggested that a separate process likely explained the sudden diffusely positive margin from a previously nearly clear one. With carefully confirmed negative margins, our patient was able to proceed with soft tissue reconstruction after a tissue sparing surgical resection.
